# Bis{tris­[3-(2-pyrid­yl)-1*H*-pyrazole]cadmium(II)} dodeca­molybdo(V,VI)phosphate hexa­hydrate

**DOI:** 10.1107/S1600536810004307

**Published:** 2010-02-06

**Authors:** Lujiang Hao, Yan Wang, Xiaofei Zhang, Jiangkui Chen, Xiutang Zhang

**Affiliations:** a College of Food and Biological Engineering, Shandong Institute of Light Industry, Jinan 250353, People’s Republic of China; b Advanced Material Institute of Research, Department of Chemistry and Chemical Engineering, ShanDong Institute of Education, Jinan 250013, People’s Republic of China; c College of Chemistry and Chemical Engineering, Liaocheng University, Liaocheng 252059, People’s Republic of China

## Abstract

The hydro­thermally prepared title compound, [Cd(C_8_H_7_N_3_)_3_]_2_[PMo_12_O_40_]·6H_2_O, is isotypic with its Mn^II^ analogue [Hao *et al.* (2010 ▶). *Acta Cryst.* E**66**, m231–m232]. The Cd^II^ cation is in a distorted octa­hedral environment, coordinated by six N atoms from three chelating 3-(2-pyrid­yl)-1*H*-pyrazole ligands. In the reduced heteropolyanion, two O atoms of the central PO_4_ group (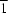 symmetry) are equally disordered about an inversion centre. N—H⋯O and O—H⋯O hydrogen bonds contribute to the crystal packing. Compared with the Mn^II^ analogue, the Cd—N bond lengths are longer at 2.316 (7)–2.334 (6) Å, *versus* 2.224 (6)–2.283 (5) Å for Mn—N, whereas all other bond lengths and angles and the hydrogen-bonding motifs are very similar in the two structures.

## Related literature

For the isotypic Mn^II^ compound, see Hao *et al.* (2010 ▶). For general background to polyoxometallates, see: Pope & Müller (1991 ▶). For polyoxometallates modified with amines, see: Zhang, Dou *et al.* (2009 ▶); Zhang, Wei *et al.* (2009 ▶). For the structures of other reduced heteropolyanions [PMo_12_O_40_]^4−^, see: Artero & Proust (2000 ▶); Kurmoo *et al.* (1998 ▶); Niu *et al.* (1999 ▶). For the role of amines in hydro­thermal synthesis, see: Yang *et al.* (2003 ▶).
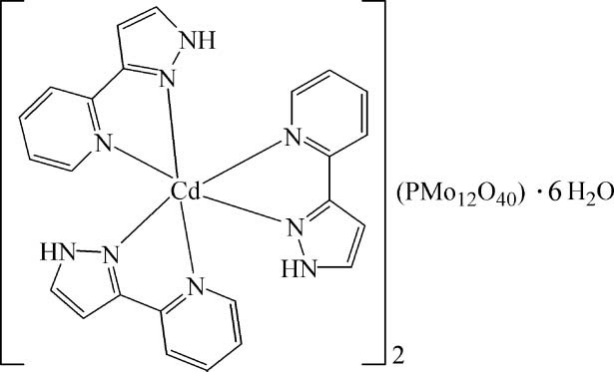


## Experimental

### 

#### Crystal data

[Cd(C_8_H_7_N_3_)_3_]_2_[PMo_12_O_40_]·6H_2_O
*M* *_r_* = 3026.14
Monoclinic, 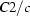

*a* = 19.0326 (14) Å
*b* = 16.4779 (12) Å
*c* = 27.267 (2) Å
β = 105.187 (1)°
*V* = 8252.8 (10) Å^3^
*Z* = 4
Mo *K*α radiation
μ = 2.39 mm^−1^
*T* = 293 K
0.12 × 0.10 × 0.08 mm

#### Data collection

Bruker APEXII CCD diffractometer
Absorption correction: multi-scan (*SADABS*; Bruker, 2001 ▶) *T*_min_ = 0.763, *T*_max_ = 0.832
21276 measured reflections
7254 independent reflections
6211 reflections with *I* > 2σ(*I*)
*R*_int_ = 0.105

#### Refinement

*R*[*F* ^2^ > 2σ(*F* ^2^)] = 0.043
*wR*(*F* ^2^) = 0.125
*S* = 1.00
7254 reflections
592 parameters
H-atom parameters constrained
Δρ_max_ = 1.55 e Å^−3^
Δρ_min_ = −0.82 e Å^−3^

### 

Data collection: *APEX2* (Bruker, 2004 ▶); cell refinement: *SAINT-Plus* (Bruker, 2001 ▶); data reduction: *SAINT-Plus*; program(s) used to solve structure: *SHELXS97* (Sheldrick, 2008 ▶); program(s) used to refine structure: *SHELXL97* (Sheldrick, 2008 ▶); molecular graphics: *SHELXTL* (Sheldrick, 2008 ▶); software used to prepare material for publication: *SHELXTL*.

## Supplementary Material

Crystal structure: contains datablocks global, I. DOI: 10.1107/S1600536810004307/wm2302sup1.cif

Structure factors: contains datablocks I. DOI: 10.1107/S1600536810004307/wm2302Isup2.hkl

Additional supplementary materials:  crystallographic information; 3D view; checkCIF report

## Figures and Tables

**Table 1:** Selected bond lengths (Å)

Cd1—N5	2.316 (7)
Cd1—N8	2.325 (7)
Cd1—N1	2.342 (6)
Cd1—N2	2.347 (6)
Cd1—N4	2.334 (7)
Cd1—N7	2.334 (6)
P1—O21*A*^i^	1.516 (8)
P1—O21*B*^i^	1.493 (8)
P1—O19*B*^i^	1.520 (7)
P1—O19*A*^i^	1.551 (7)

Symmetry code: (i) 
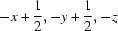
.

**Table 2:** Hydrogen-bond geometry (Å, °)

*D*—H⋯*A*	*D*—H	H⋯*A*	*D*⋯*A*	*D*—H⋯*A*
N3—H3*A*⋯O17^ii^	0.86	1.95	2.803 (8)	171
N6—H6⋯O2*W*	0.86	2.25	3.102 (17)	173
N9—H9*A*⋯O1*W*	0.86	1.96	2.790 (11)	163

Symmetry code: (ii) 
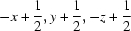
.

**Fig. 1.**
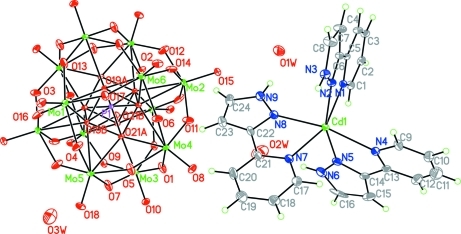
The building blocks of the title compound with the atom-labelling scheme. Displacement ellipsoids are drawn at the 30% probability level; H atoms are given as spheres of arbitrary radius.

**Fig. 2.**
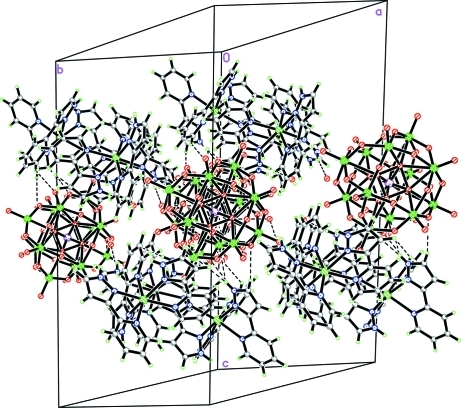
The crystal packing of the title compound, displayed with N—H···O and O—H···O hydrogen bonds as dashed lines.

## supplementary crystallographic information

### Comment

The design and synthesis of polyoxometalates has attracted continuous research interest not only because of their appealing structural and topological novelties, but also due to their interesting optical, electronic, magnetic, and catalytic properties, as well as their potential medical applications (Pope & Müller, 1991). In our research group, organic amines, such as 3-(2-pyridyl)pyrazole and pyrazine, are used to effectively modify polyoxomolybdates under hydrothermal condictions (Zhang, Dou *et al.*, 2009; Zhang, Wei *et al.*, 2009). Here, we describe the synthesis and structural characterization of the title compound.

As shown in Fig. 1, the asymmetric unit of the title compound consists of three subunits, *viz.* of a complex [Cd(C_8_H_7_N_3_)_3_]^2+^ cation, half of a a [PMo_12_O_40_]^4-^ heteropolyanion and of three uncoordinated water molecules. The cadmium(II) ion is distorted octahedrally coordinated by six N atoms from three chelating 3-(2-pyridyl)-1*H*-pyrazole ligands.

The heteropolyanion [PMo_12_O_40_]^4-^ anion is an one-electron reduced derivative of [PMo_12_O_40_]^3-^, similar to anions with different counter cations as reported by Artero & Proust (2000); Kurmoo *et al.* (1998); Niu *et al.* (1999). The employed organic ligand appears to adjust the pH value, and additionally supplies reducing electrons, which is a commonly observed feature of hydrothermal syntheses when organic amines are used to prepare various hybrid materials, zeolites or metal phosphates (Yang *et al.*, 2003).

In the Keggin-type heteropolyanion, each Mo atom is surrounded by six O atoms and the P atom is located at the center of the anion. There are four kinds of O atoms present in the anion according to their coordination environments: O*_a_* (O atoms in the disordered PO_4_ tetrahedron), O*_b_* (bridging O atoms between two triplet groups of MoO_6_ octahedra), O*_c_* (bridging O atoms within one triplet group of MoO_6_ octahedra) and O*_d_* (terminal O atoms). The P—O bond distances are in the normal range of 1.516 (8)–1.551 (7) Å. The Mo—O bond distances vary widely from 1.637 (4) to 2.509 (7) Å. The shortest Mo—O bonds are in the range of 1.637 (4)–1.661 (4) Å for the terminal oxygen atoms. The longest Mo—O lengths are in the range of 2.420 (7)–2.509 (7) Å for those oxygen atoms connected with both Mo and P atoms.

N—H···O and O—H···O hydrogen bonding between the cationic and anionic moieties and the uncoordinated water molecules leads to a consolidation of the structure (Fig. 2; Table 2).

The crystal structure of [(Cd(C_8_H_7_N_3_)_3_]_2_[PMo_12_O_40_](H_2_O)_6_ is isotypic with the Mn^2+^ analogue, [(Mn(C_8_H_7_N_3_)_3_]_2_[PMo_12_O_40_](H_2_O)_6_ (Hao *et al.*, 2010). In comparison with the Mn(II) analogue, the Cd—N bond lengths are longer, 2.316 (7)–2.334 (6) Å *versus* 2.224 (6)–2.283 (5) Å for Mn—N, whereas all other bond lengths, angles and the hydrogen bonding motifs are very similar in the two structures.

### Experimental

A mixture of 3-(2-pyridyl)-1*H*-pyrazole (0.5 mmoL 0.07 g), sodium molybdate (0.4 mmoL, 0.10 g), cadmium sulfate (0.25 mmol, 0.04 g), and dipotassium hydrogenphosphate (0.22 mmol, 0.05 g) in 10 ml distilled water was sealed in a 25 ml Teflon-lined stainless steel autoclave and was kept at 433 K for three days. Colorless crystals suitable for the X-ray experiment were obtained. Anal. C_48_H_54_Cd_2_Mo_12_N_18_O_46_P: C, 19.05; H, 1.80; N, 8.33. Found: C, 18.95; H, 1.62; N, 7.03 %. IR(cm^-1^): 3312, 3136, 1604, 1457, 1503, 1429, 1097, 1060, 950, 811, 765, 590, 498, 405.

TGA curve shows a separation of lattice water molecules and the organic ligands above 350 and 588 K, respectively. The overall thermal decomposition process can be described by the followed equation: 4C_48_H_54_Cd_2_Mo_12_N_18_O_46_P + 325O_2_ = 108H_2_O + 192CO_2_ + 36N_2_O_5_ + 8CdO + 2P_2_O_5_ + 48MoO_3_

### Refinement

All hydrogen atoms bound to aromatic carbon atoms were refined in calculated positions using a riding model with a C—H distance of 0.93 Å and *U*_iso_ = 1.2*U*_eq_(C). Hydrogen atoms attached to aromatic N atoms were refined with a N—H distance of 0.86 Å and *U*_iso_ = 1.2*U*_eq_(N). The hydrogen atoms of the three uncoordinated water molecules could not be located unambiguously from difference Fourier maps, probably due to disorder of the water molecules. Thus the structure was refined without the H atoms of the water molecules (which includes the water O atoms O1W, O2W, O3W). In the PO_4_ unit, the two oxygen atoms (O19 and O21) are equally disordered about the inversion centre. In the final difference Fourier map the highest peak is 2.70 Å from atom O2w and the deepest hole is 0.46 Å from atom Mo1. The highest peak is located in the voids of the crystal structure and may be associated with an additional water molecule. However, refinement of this position did not result in a reasonable model. Hence this position was also excluded from the final refinement.

### Crystal data

**Table:**

[Cd(C_8_H_7_N_3_)_3_]_2_[PMo_12_O_40_]·6H_2_O	*F*(000) = 5804
*M**_r_* = 3026.14	*D*_x_ = 2.436 Mg m^−^^3^
Monoclinic, *C*2/*c*	Mo *K*α radiation, λ = 0.71073 Å
Hall symbol: -C 2yc	Cell parameters from 5617 reflections
*a* = 19.0326 (14) Å	θ = 2.5–28.4°
*b* = 16.4779 (12) Å	µ = 2.38 mm^−^^1^
*c* = 27.267 (2) Å	*T* = 293 K
β = 105.187 (1)°	Block, colourless
*V* = 8252.8 (10) Å^3^	0.12 × 0.10 × 0.08 mm
*Z* = 4	

### Data collection

**Table:**

Bruker APEXII CCD diffractometer	7254 independent reflections
Radiation source: fine-focus sealed tube	6211 reflections with *I* > 2σ(*I*)
graphite	*R*_int_ = 0.105
φ and ω scans	θ_max_ = 25.0°, θ_min_ = 1.7°
Absorption correction: multi-scan (*SADABS*; Bruker, 2001)	*h* = −22→22
*T*_min_ = 0.763, *T*_max_ = 0.832	*k* = −19→13
21276 measured reflections	*l* = −32→32

### Refinement

**Table:**

Refinement on *F*^2^	Primary atom site location: structure-invariant direct methods
Least-squares matrix: full	Secondary atom site location: difference Fourier map
*R*[*F*^2^ > 2σ(*F*^2^)] = 0.043	Hydrogen site location: inferred from neighbouring sites
*wR*(*F*^2^) = 0.125	H-atom parameters constrained
*S* = 1.00	*w* = 1/[σ^2^(*F*_o_^2^) + (0.057*P*)^2^ + 46.070*P*] where *P* = (*F*_o_^2^ + 2*F*_c_^2^)/3
7254 reflections	(Δ/σ)_max_ = 0.001
592 parameters	Δρ_max_ = 1.55 e Å^−^^3^
0 restraints	Δρ_min_ = −0.82 e Å^−^^3^

### Special details

**Table:**

Geometry. All esds (except the esd in the dihedral angle between two l.s. planes) are estimated using the full covariance matrix. The cell esds are taken into account individually in the estimation of esds in distances, angles and torsion angles; correlations between esds in cell parameters are only used when they are defined by crystal symmetry. An approximate (isotropic) treatment of cell esds is used for estimating esds involving l.s. planes.
Refinement. Refinement of *F*^2^ against ALL reflections. The weighted *R*-factor wR and goodness of fit *S* are based on *F*^2^, conventional *R*-factors *R* are based on *F*, with *F* set to zero for negative *F*^2^. The threshold expression of *F*^2^ > σ(*F*^2^) is used only for calculating *R*-factors(gt) etc. and is not relevant to the choice of reflections for refinement. *R*-factors based on *F*^2^ are statistically about twice as large as those based on *F*, and *R*- factors based on ALL data will be even larger.

### Fractional atomic coordinates and isotropic or equivalent isotropic displacement parameters (Å^2^)

**Table:**

	*x*	*y*	*z*	*U*_iso_*/*U*_eq_	Occ. (<1)
C1	0.2003 (5)	0.9040 (4)	0.0936 (3)	0.0523 (18)	
H1	0.2436	0.9008	0.0841	0.063*	
C2	0.1407 (5)	0.9372 (5)	0.0604 (3)	0.055 (2)	
H2	0.1437	0.9559	0.0289	0.065*	
C3	0.0772 (4)	0.9426 (5)	0.0738 (3)	0.0529 (19)	
H3	0.0363	0.9655	0.0517	0.063*	
C4	0.0741 (4)	0.9138 (5)	0.1202 (2)	0.0501 (18)	
H4	0.0308	0.9162	0.1301	0.060*	
C5	0.1369 (4)	0.8809 (4)	0.1527 (2)	0.0371 (14)	
C6	0.1383 (4)	0.8536 (4)	0.2040 (2)	0.0416 (15)	
C7	0.0812 (5)	0.8491 (7)	0.2271 (3)	0.070 (3)	
H7	0.0327	0.8633	0.2136	0.084*	
C8	0.1136 (6)	0.8186 (7)	0.2744 (3)	0.078 (3)	
H8	0.0903	0.8076	0.2997	0.094*	
C9	0.3372 (5)	0.9934 (5)	0.2687 (3)	0.061 (2)	
H9	0.3085	0.9745	0.2891	0.073*	
C10	0.3625 (6)	1.0678 (6)	0.2741 (3)	0.072 (3)	
H10	0.3494	1.1012	0.2977	0.087*	
C11	0.4017 (9)	1.0952 (10)	0.2501 (6)	0.125 (5)	
H11	0.4196	1.1477	0.2570	0.150*	
C12	0.4183 (7)	1.0575 (7)	0.2181 (5)	0.093 (4)	
H12	0.4480	1.0809	0.1999	0.112*	
C13	0.3945 (4)	0.9806 (5)	0.2078 (3)	0.055 (2)	
C14	0.4148 (4)	0.9338 (6)	0.1693 (3)	0.058 (2)	
C15	0.4670 (6)	0.9529 (8)	0.1422 (4)	0.094 (3)	
H15	0.4966	0.9985	0.1451	0.113*	
C16	0.4624 (6)	0.8789 (11)	0.1064 (4)	0.132 (6)	
H16	0.4884	0.8698	0.0824	0.158*	
C17	0.4153 (5)	0.7420 (6)	0.2994 (3)	0.066 (2)	
H17	0.4208	0.7957	0.3102	0.079*	
C18	0.4552 (6)	0.6849 (7)	0.3306 (4)	0.084 (3)	
H18	0.4860	0.6987	0.3620	0.100*	
C19	0.4482 (7)	0.6075 (7)	0.3142 (4)	0.097 (3)	
H19	0.4756	0.5670	0.3341	0.116*	
C20	0.4011 (7)	0.5879 (6)	0.2683 (4)	0.088 (3)	
H20	0.3962	0.5345	0.2569	0.106*	
C21	0.3613 (4)	0.6494 (5)	0.2394 (3)	0.0510 (18)	
C22	0.3078 (5)	0.6333 (5)	0.1910 (3)	0.0537 (19)	
C23	0.2869 (6)	0.5616 (6)	0.1655 (3)	0.070 (2)	
H23	0.3043	0.5099	0.1757	0.084*	
C24	0.2367 (7)	0.5815 (6)	0.1233 (4)	0.082 (3)	
H24	0.2127	0.5459	0.0979	0.099*	
Cd1	0.29910 (3)	0.82317 (3)	0.19989 (2)	0.04993 (16)	
Mo1	0.24356 (3)	0.14473 (4)	0.112775 (19)	0.03963 (16)	
Mo2	0.18969 (3)	0.45389 (3)	−0.01844 (2)	0.03810 (16)	
Mo3	0.42042 (3)	0.31320 (4)	−0.01864 (2)	0.03638 (15)	
Mo4	0.35147 (3)	0.40777 (3)	0.07580 (2)	0.03486 (15)	
Mo5	0.41452 (3)	0.20314 (4)	0.09234 (2)	0.03702 (16)	
Mo6	0.17953 (3)	0.34705 (4)	0.09257 (2)	0.03701 (16)	
N1	0.1987 (3)	0.8760 (3)	0.1393 (2)	0.0404 (13)	
N2	0.1995 (3)	0.8305 (4)	0.2354 (2)	0.0449 (14)	
N3	0.1831 (4)	0.8077 (4)	0.2780 (2)	0.0535 (17)	
H3A	0.2139	0.7885	0.3044	0.064*	
N4	0.3528 (4)	0.9451 (4)	0.2340 (3)	0.0599 (17)	
N5	0.3811 (4)	0.8630 (5)	0.1550 (3)	0.0623 (18)	
N6	0.4094 (5)	0.8280 (6)	0.1188 (3)	0.088 (3)	
H6	0.3967	0.7813	0.1054	0.106*	
N7	0.3687 (4)	0.7268 (4)	0.2543 (3)	0.0540 (16)	
N8	0.2707 (4)	0.6949 (4)	0.1647 (2)	0.0558 (16)	
N9	0.2269 (4)	0.6618 (4)	0.1238 (3)	0.0661 (19)	
H9A	0.1962	0.6884	0.1006	0.079*	
O1	0.4052 (4)	0.3934 (3)	0.02395 (19)	0.0642 (16)	
O2	0.1518 (3)	0.3937 (4)	0.13815 (18)	0.0588 (14)	
O3	0.2429 (3)	0.0954 (3)	0.16477 (17)	0.0559 (14)	
O4	0.3462 (3)	0.1774 (4)	0.1271 (2)	0.0707 (17)	
O5	0.4032 (4)	0.3144 (3)	0.1061 (2)	0.0695 (17)	
O6	0.2803 (3)	0.3886 (4)	0.1088 (2)	0.0637 (15)	
O7	0.4496 (4)	0.2421 (3)	0.0358 (2)	0.0658 (16)	
O8	0.4017 (3)	0.4809 (3)	0.11005 (19)	0.0580 (14)	
O9	0.3983 (4)	0.2149 (3)	−0.0612 (2)	0.084 (2)	
O10	0.4989 (3)	0.3398 (3)	−0.0294 (2)	0.0556 (13)	
O11	0.2845 (3)	0.4671 (4)	0.0255 (2)	0.0693 (16)	
O12	0.1054 (3)	0.3955 (4)	−0.0555 (2)	0.076 (2)	
O13	0.1468 (4)	0.1374 (3)	0.0742 (3)	0.084 (2)	
O14	0.1593 (3)	0.4231 (4)	0.0411 (2)	0.0680 (16)	
O15	0.1625 (3)	0.5493 (3)	−0.0258 (2)	0.0643 (15)	
O16	0.2700 (4)	0.0629 (4)	0.0726 (2)	0.085 (2)	
O17	0.2257 (3)	0.2543 (3)	0.1295 (2)	0.0758 (18)	
O18	0.4915 (3)	0.1816 (4)	0.13433 (18)	0.0575 (14)	
O19A	0.2060 (4)	0.2389 (5)	0.0400 (3)	0.0249 (16)	0.50
O21A	0.3254 (4)	0.2809 (5)	0.0264 (3)	0.0286 (17)	0.50
O19B	0.2893 (4)	0.1874 (4)	0.0384 (3)	0.0269 (16)	0.50
O21B	0.2466 (4)	0.3295 (5)	0.0256 (3)	0.0283 (17)	0.50
O1W	0.1149 (5)	0.7162 (5)	0.0426 (3)	0.107 (3)	
O2W	0.3647 (8)	0.6659 (10)	0.0613 (6)	0.207 (7)	
O3W	0.5313 (8)	0.0724 (8)	0.0267 (7)	0.220 (8)	
P1	0.2500	0.2500	0.0000	0.0216 (4)	

### Atomic displacement parameters (Å^2^)

**Table:**

	*U*^11^	*U*^22^	*U*^33^	*U*^12^	*U*^13^	*U*^23^
C1	0.059 (5)	0.049 (4)	0.052 (4)	−0.001 (4)	0.022 (4)	0.016 (3)
C2	0.065 (6)	0.054 (4)	0.042 (4)	−0.013 (4)	0.010 (4)	0.013 (4)
C3	0.041 (4)	0.059 (5)	0.047 (4)	−0.006 (4)	−0.008 (3)	0.011 (4)
C4	0.035 (4)	0.075 (5)	0.039 (4)	−0.001 (4)	0.006 (3)	0.006 (4)
C5	0.032 (4)	0.038 (3)	0.040 (3)	−0.003 (3)	0.008 (3)	0.000 (3)
C6	0.034 (4)	0.054 (4)	0.036 (3)	0.002 (3)	0.008 (3)	0.004 (3)
C7	0.047 (5)	0.117 (8)	0.050 (5)	0.014 (5)	0.021 (4)	0.019 (5)
C8	0.069 (7)	0.124 (9)	0.050 (5)	0.015 (6)	0.030 (4)	0.025 (5)
C9	0.077 (6)	0.051 (5)	0.066 (5)	0.010 (4)	0.040 (4)	0.018 (4)
C10	0.083 (7)	0.071 (6)	0.053 (5)	−0.002 (5)	0.001 (4)	−0.001 (5)
C11	0.118 (9)	0.118 (9)	0.122 (8)	0.001 (7)	0.002 (7)	0.010 (7)
C12	0.087 (8)	0.080 (7)	0.086 (7)	−0.020 (6)	−0.026 (6)	0.035 (6)
C13	0.033 (4)	0.055 (5)	0.067 (5)	0.000 (4)	−0.005 (3)	0.020 (4)
C14	0.033 (4)	0.071 (5)	0.065 (5)	−0.001 (4)	0.003 (3)	0.028 (4)
C15	0.054 (6)	0.122 (7)	0.099 (6)	−0.014 (5)	0.006 (5)	0.051 (6)
C16	0.062 (6)	0.288 (18)	0.060 (6)	0.104 (9)	0.043 (5)	0.069 (9)
C17	0.065 (6)	0.066 (5)	0.058 (5)	0.002 (5)	0.001 (4)	0.002 (4)
C18	0.075 (7)	0.091 (8)	0.073 (6)	0.001 (6)	−0.001 (5)	0.015 (6)
C19	0.090 (7)	0.086 (6)	0.098 (6)	0.007 (5)	−0.003 (5)	0.037 (6)
C20	0.109 (9)	0.052 (5)	0.091 (7)	0.019 (5)	0.004 (6)	0.017 (5)
C21	0.052 (5)	0.047 (4)	0.059 (5)	0.015 (4)	0.024 (4)	0.012 (4)
C22	0.063 (5)	0.044 (4)	0.060 (5)	0.015 (4)	0.026 (4)	0.004 (4)
C23	0.089 (7)	0.056 (5)	0.063 (5)	0.013 (5)	0.018 (5)	−0.001 (4)
C24	0.115 (9)	0.061 (6)	0.073 (6)	0.001 (6)	0.029 (6)	−0.023 (5)
Cd1	0.0367 (3)	0.0416 (3)	0.0716 (4)	0.0045 (2)	0.0144 (3)	0.0096 (3)
Mo1	0.0479 (4)	0.0429 (3)	0.0267 (3)	−0.0076 (3)	0.0072 (2)	0.0037 (2)
Mo2	0.0380 (3)	0.0301 (3)	0.0478 (3)	0.0041 (2)	0.0139 (2)	−0.0001 (2)
Mo3	0.0245 (3)	0.0445 (3)	0.0420 (3)	−0.0052 (2)	0.0119 (2)	−0.0008 (2)
Mo4	0.0295 (3)	0.0380 (3)	0.0363 (3)	−0.0073 (2)	0.0071 (2)	−0.0097 (2)
Mo5	0.0251 (3)	0.0505 (3)	0.0324 (3)	0.0016 (2)	0.0020 (2)	0.0006 (2)
Mo6	0.0360 (3)	0.0450 (3)	0.0312 (3)	0.0049 (2)	0.0107 (2)	−0.0108 (2)
N1	0.037 (3)	0.042 (3)	0.044 (3)	−0.001 (3)	0.014 (2)	0.006 (3)
N2	0.040 (3)	0.055 (4)	0.038 (3)	0.002 (3)	0.007 (2)	0.008 (3)
N3	0.055 (4)	0.067 (4)	0.035 (3)	0.001 (3)	0.006 (3)	0.013 (3)
N4	0.046 (4)	0.049 (4)	0.087 (5)	−0.002 (3)	0.022 (4)	0.006 (4)
N5	0.042 (4)	0.070 (5)	0.080 (5)	0.011 (3)	0.026 (3)	0.013 (4)
N6	0.076 (6)	0.094 (6)	0.089 (6)	0.027 (5)	0.012 (5)	−0.009 (5)
N7	0.048 (4)	0.052 (4)	0.062 (4)	0.005 (3)	0.015 (3)	0.008 (3)
N8	0.053 (4)	0.054 (4)	0.057 (4)	0.004 (3)	0.008 (3)	−0.004 (3)
N9	0.068 (5)	0.066 (4)	0.061 (4)	0.006 (4)	0.010 (4)	−0.004 (4)
O1	0.098 (4)	0.057 (3)	0.051 (3)	0.032 (3)	0.043 (3)	0.019 (2)
O2	0.046 (3)	0.091 (4)	0.046 (3)	0.002 (3)	0.023 (2)	−0.026 (3)
O3	0.058 (3)	0.073 (4)	0.034 (2)	−0.016 (3)	0.006 (2)	0.013 (2)
O4	0.068 (4)	0.080 (4)	0.081 (4)	−0.038 (3)	0.047 (3)	−0.041 (3)
O5	0.106 (5)	0.058 (3)	0.064 (3)	0.032 (3)	0.057 (3)	0.017 (3)
O6	0.037 (3)	0.096 (4)	0.058 (3)	0.000 (3)	0.014 (2)	0.030 (3)
O7	0.103 (4)	0.055 (3)	0.056 (3)	0.032 (3)	0.050 (3)	0.012 (3)
O8	0.061 (4)	0.055 (3)	0.051 (3)	−0.024 (3)	0.004 (2)	−0.009 (3)
O9	0.081 (5)	0.042 (3)	0.090 (4)	0.010 (3)	−0.047 (4)	−0.019 (3)
O10	0.040 (3)	0.058 (3)	0.079 (3)	−0.007 (2)	0.033 (3)	−0.010 (3)
O11	0.038 (3)	0.111 (4)	0.060 (3)	0.006 (3)	0.015 (2)	0.037 (3)
O12	0.069 (4)	0.094 (5)	0.083 (4)	−0.043 (4)	0.051 (3)	−0.052 (4)
O13	0.080 (5)	0.040 (3)	0.095 (4)	0.006 (3)	−0.044 (4)	−0.017 (3)
O14	0.032 (3)	0.112 (4)	0.058 (3)	−0.001 (3)	0.008 (2)	0.029 (3)
O15	0.052 (4)	0.039 (3)	0.099 (4)	0.010 (2)	0.015 (3)	0.008 (3)
O16	0.074 (4)	0.106 (5)	0.094 (4)	−0.059 (4)	0.056 (4)	−0.062 (4)
O17	0.067 (4)	0.045 (3)	0.083 (4)	0.002 (3)	−0.038 (3)	−0.014 (3)
O18	0.042 (3)	0.078 (4)	0.044 (3)	0.017 (3)	−0.003 (2)	0.002 (3)
O19A	0.024 (4)	0.029 (4)	0.021 (4)	−0.003 (3)	0.005 (3)	−0.011 (3)
O21A	0.023 (4)	0.034 (4)	0.029 (4)	−0.006 (3)	0.008 (3)	−0.004 (3)
O19B	0.027 (4)	0.024 (4)	0.030 (4)	−0.003 (3)	0.007 (3)	0.001 (3)
O21B	0.022 (4)	0.035 (4)	0.029 (4)	−0.002 (3)	0.009 (3)	−0.001 (3)
O1W	0.086 (6)	0.108 (6)	0.110 (6)	0.018 (5)	−0.005 (4)	0.008 (5)
O2W	0.170 (14)	0.254 (16)	0.239 (14)	−0.062 (11)	0.126 (12)	−0.079 (12)
O3W	0.199 (14)	0.149 (10)	0.39 (2)	−0.012 (9)	0.210 (15)	−0.082 (12)
P1	0.0184 (10)	0.0271 (10)	0.0191 (9)	−0.0006 (8)	0.0046 (7)	−0.0017 (8)

### Geometric parameters (Å, °)

**Table:**

C1—N1	1.334 (8)	Mo1—O19B	2.509 (7)
C1—C2	1.366 (11)	Mo2—O15	1.650 (5)
C1—H1	0.9300	Mo2—O16^i^	1.854 (5)
C2—C3	1.352 (12)	Mo2—O11	1.900 (6)
C2—H2	0.9300	Mo2—O14	1.927 (5)
C3—C4	1.369 (10)	Mo2—O12	1.916 (6)
C3—H3	0.9300	Mo2—O21B	2.477 (8)
C4—C5	1.398 (10)	Mo2—O19B^i^	2.447 (7)
C4—H4	0.9300	Mo3—O10	1.655 (5)
C5—N1	1.324 (8)	Mo3—O1	1.831 (5)
C5—C6	1.462 (9)	Mo3—O7	1.858 (5)
C6—N2	1.310 (9)	Mo3—O13^i^	1.892 (6)
C6—C7	1.392 (11)	Mo3—O9	1.972 (5)
C7—C8	1.372 (12)	Mo3—O19A^i^	2.476 (7)
C7—H7	0.9300	Mo3—O21A	2.496 (7)
C8—N3	1.313 (11)	Mo4—O8	1.664 (5)
C8—H8	0.9300	Mo4—O6	1.840 (5)
C9—N4	1.328 (10)	Mo4—O11	1.882 (6)
C9—C10	1.312 (13)	Mo4—O5	1.896 (5)
C9—H9	0.9300	Mo4—O1	1.964 (5)
C10—C11	1.201 (18)	Mo4—O21B	2.468 (8)
C10—H10	0.9300	Mo4—O21A	2.466 (8)
C11—C12	1.179 (18)	Mo5—O18	1.645 (5)
C11—H11	0.9300	Mo5—O4	1.849 (5)
C12—C13	1.350 (13)	Mo5—O5	1.894 (5)
C12—H12	0.9300	Mo5—O12^i^	1.897 (5)
C13—N4	1.335 (10)	Mo5—O7	1.943 (5)
C13—C14	1.434 (12)	Mo5—O19B	2.464 (8)
C14—N5	1.340 (11)	Mo5—O21A	2.480 (8)
C14—C15	1.421 (13)	Mo6—O2	1.661 (4)
C15—C16	1.549 (19)	Mo6—O9^i^	1.818 (6)
C15—H15	0.9300	Mo6—O14	1.847 (5)
C16—N6	1.419 (17)	Mo6—O17	1.913 (6)
C16—H16	0.9300	Mo6—O6	1.975 (6)
C17—N7	1.338 (11)	Mo6—O19A	2.420 (7)
C17—C18	1.359 (13)	Mo6—O21B	2.505 (7)
C17—H17	0.9300	N2—N3	1.334 (8)
C18—C19	1.346 (15)	N3—H3A	0.8600
C18—H18	0.9300	N5—N6	1.368 (11)
C19—C20	1.373 (15)	N6—H6	0.8600
C19—H19	0.9300	N8—N9	1.323 (9)
C20—C21	1.381 (11)	N9—H9A	0.8600
C20—H20	0.9300	O9—Mo6^i^	1.818 (6)
C21—N7	1.335 (10)	O12—Mo5^i^	1.897 (5)
C21—C22	1.464 (12)	O13—Mo3^i^	1.892 (6)
C22—N8	1.333 (10)	O16—Mo2^i^	1.854 (5)
C22—C23	1.376 (12)	O19A—P1	1.551 (7)
C23—C24	1.330 (14)	O19A—O21A^i^	1.784 (10)
C23—H23	0.9300	O19A—Mo3^i^	2.476 (7)
C24—N9	1.336 (11)	O21A—P1	1.516 (8)
C24—H24	0.9300	O21A—O21B	1.695 (11)
Cd1—N5	2.316 (7)	O21A—O19A^i^	1.784 (10)
Cd1—N8	2.325 (7)	O21A—O19B	1.752 (10)
Cd1—N1	2.342 (6)	O19B—P1	1.520 (7)
Cd1—N2	2.347 (6)	O19B—O21B^i^	1.721 (10)
Cd1—N4	2.334 (7)	O19B—Mo2^i^	2.447 (7)
Cd1—N7	2.334 (6)	O21B—P1	1.493 (8)
Mo1—O3	1.637 (4)	O21B—O19B^i^	1.721 (10)
Mo1—O13	1.871 (6)	P1—O21A^i^	1.516 (8)
Mo1—O16	1.888 (6)	P1—O21B^i^	1.493 (8)
Mo1—O17	1.914 (6)	P1—O19B^i^	1.520 (7)
Mo1—O4	1.964 (6)	P1—O19A^i^	1.551 (7)
Mo1—O19A	2.472 (7)		
			
N1—C1—C2	122.6 (7)	O18—Mo5—O4	102.2 (3)
N1—C1—H1	118.6	O18—Mo5—O5	101.4 (3)
C2—C1—H1	118.7	O4—Mo5—O5	89.3 (3)
C1—C2—C3	119.5 (7)	O18—Mo5—O12^i^	101.6 (3)
C1—C2—H2	120.5	O4—Mo5—O12^i^	90.3 (2)
C3—C2—H2	120.0	O5—Mo5—O12^i^	156.5 (3)
C4—C3—C2	119.0 (7)	O18—Mo5—O7	101.2 (3)
C4—C3—H3	120.4	O4—Mo5—O7	156.6 (3)
C2—C3—H3	120.6	O5—Mo5—O7	85.3 (2)
C3—C4—C5	118.9 (7)	O12^i^—Mo5—O7	85.7 (2)
C3—C4—H4	120.6	O18—Mo5—O19B	159.9 (3)
C5—C4—H4	120.5	O4—Mo5—O19B	65.3 (3)
N1—C5—C4	121.5 (6)	O5—Mo5—O19B	94.5 (3)
N1—C5—C6	116.9 (6)	O12^i^—Mo5—O19B	64.3 (3)
C4—C5—C6	121.5 (6)	O7—Mo5—O19B	92.3 (3)
N2—C6—C7	110.8 (6)	O18—Mo5—O21A	158.6 (3)
N2—C6—C5	120.3 (6)	O4—Mo5—O21A	92.9 (3)
C7—C6—C5	128.9 (7)	O5—Mo5—O21A	63.4 (3)
C8—C7—C6	103.5 (8)	O12^i^—Mo5—O21A	93.2 (3)
C8—C7—H7	128.3	O7—Mo5—O21A	64.3 (2)
C6—C7—H7	128.3	O19B—Mo5—O21A	41.5 (2)
N3—C8—C7	108.4 (7)	O2—Mo6—O9^i^	103.5 (3)
N3—C8—H8	125.8	O2—Mo6—O14	102.2 (3)
C7—C8—H8	125.7	O9^i^—Mo6—O14	92.4 (3)
N4—C9—C10	119.7 (8)	O2—Mo6—O17	99.7 (3)
N4—C9—H9	120.0	O9^i^—Mo6—O17	90.3 (2)
C10—C9—H9	120.3	O14—Mo6—O17	156.7 (3)
C11—C10—C9	123.6 (13)	O2—Mo6—O6	98.8 (3)
C11—C10—H10	118.7	O9^i^—Mo6—O6	157.5 (3)
C9—C10—H10	117.7	O14—Mo6—O6	86.1 (2)
C12—C11—C10	121.9 (18)	O17—Mo6—O6	82.7 (3)
C12—C11—H11	119.3	O2—Mo6—O19A	160.0 (3)
C10—C11—H11	118.8	O9^i^—Mo6—O19A	65.0 (3)
C11—C12—C13	120.6 (15)	O14—Mo6—O19A	94.8 (3)
C11—C12—H12	119.6	O17—Mo6—O19A	65.5 (3)
C13—C12—H12	119.8	O6—Mo6—O19A	92.7 (3)
N4—C13—C12	120.9 (10)	O2—Mo6—O21B	157.2 (3)
N4—C13—C14	117.9 (7)	O9^i^—Mo6—O21B	95.7 (3)
C12—C13—C14	121.2 (10)	O14—Mo6—O21B	64.4 (3)
N5—C14—C15	112.8 (9)	O17—Mo6—O21B	92.3 (3)
N5—C14—C13	118.7 (7)	O6—Mo6—O21B	63.5 (2)
C15—C14—C13	128.4 (9)	O19A—Mo6—O21B	42.1 (3)
C14—C15—C16	102.8 (9)	C1—N1—C5	118.5 (6)
C14—C15—H15	129.2	C1—N1—Cd1	125.0 (5)
C16—C15—H15	128.0	C5—N1—Cd1	116.5 (4)
N6—C16—C15	104.0 (7)	C6—N2—N3	106.2 (6)
N6—C16—H16	127.6	C6—N2—Cd1	115.0 (4)
C15—C16—H16	128.4	N3—N2—Cd1	138.1 (5)
N7—C17—C18	125.0 (9)	C8—N3—N2	111.0 (6)
N7—C17—H17	117.6	C8—N3—H3A	124.5
C18—C17—H17	117.5	N2—N3—H3A	124.5
C17—C18—C19	117.2 (10)	C13—N4—C9	113.2 (7)
C17—C18—H18	121.3	C13—N4—Cd1	115.1 (6)
C19—C18—H18	121.5	C9—N4—Cd1	130.1 (6)
C20—C19—C18	120.6 (10)	C14—N5—N6	109.1 (7)
C20—C19—H19	119.6	C14—N5—Cd1	115.5 (6)
C18—C19—H19	119.8	N6—N5—Cd1	135.1 (6)
C19—C20—C21	118.5 (10)	N5—N6—C16	111.2 (9)
C19—C20—H20	120.7	N5—N6—H6	124.1
C21—C20—H20	120.8	C16—N6—H6	124.7
N7—C21—C20	121.9 (8)	C17—N7—C21	116.7 (7)
N7—C21—C22	116.1 (7)	C17—N7—Cd1	125.9 (6)
C20—C21—C22	122.0 (8)	C21—N7—Cd1	117.4 (5)
N8—C22—C23	109.9 (7)	N9—N8—C22	105.5 (7)
N8—C22—C21	119.4 (7)	N9—N8—Cd1	138.5 (5)
C23—C22—C21	130.7 (7)	C22—N8—Cd1	116.0 (5)
C24—C23—C22	105.8 (8)	N8—N9—C24	111.0 (8)
C24—C23—H23	127.1	N8—N9—H9A	124.5
C22—C23—H23	127.0	C24—N9—H9A	124.4
N9—C24—C23	107.8 (9)	Mo3—O1—Mo4	137.9 (3)
N9—C24—H24	126.2	Mo5—O4—Mo1	139.2 (3)
C23—C24—H24	126.0	Mo5—O5—Mo4	140.5 (4)
N5—Cd1—N8	99.0 (3)	Mo4—O6—Mo6	138.2 (3)
N5—Cd1—N1	93.4 (2)	Mo3—O7—Mo5	138.5 (3)
N8—Cd1—N1	89.4 (2)	Mo6^i^—O9—Mo3	139.5 (4)
N5—Cd1—N2	158.8 (2)	Mo4—O11—Mo2	138.6 (4)
N8—Cd1—N2	95.2 (2)	Mo5^i^—O12—Mo2	137.1 (4)
N1—Cd1—N2	71.01 (19)	Mo1—O13—Mo3^i^	141.4 (4)
N5—Cd1—N4	71.4 (3)	Mo6—O14—Mo2	140.4 (4)
N8—Cd1—N4	167.7 (2)	Mo2^i^—O16—Mo1	142.9 (4)
N1—Cd1—N4	98.7 (2)	Mo1—O17—Mo6	135.6 (3)
N2—Cd1—N4	96.2 (2)	P1—O19A—O21A^i^	53.5 (3)
N5—Cd1—N7	100.3 (2)	P1—O19A—Mo6	124.4 (4)
N8—Cd1—N7	71.1 (2)	O21A^i^—O19A—Mo6	132.0 (5)
N1—Cd1—N7	157.5 (2)	P1—O19A—Mo3^i^	123.0 (4)
N2—Cd1—N7	99.1 (2)	O21A^i^—O19A—Mo3^i^	69.5 (3)
N4—Cd1—N7	102.5 (2)	Mo6—O19A—Mo3^i^	93.1 (3)
O3—Mo1—O13	103.0 (3)	P1—O19A—Mo1	122.9 (4)
O3—Mo1—O16	102.2 (3)	O21A^i^—O19A—Mo1	130.5 (5)
O13—Mo1—O16	90.0 (3)	Mo6—O19A—Mo1	92.8 (2)
O3—Mo1—O17	102.7 (3)	Mo3^i^—O19A—Mo1	91.7 (2)
O13—Mo1—O17	89.1 (2)	P1—O21A—O21B	55.1 (4)
O16—Mo1—O17	154.6 (3)	P1—O21A—O19A^i^	55.4 (3)
O3—Mo1—O4	101.3 (3)	O21B—O21A—O19A^i^	90.7 (5)
O13—Mo1—O4	155.7 (3)	P1—O21A—O19B	54.9 (4)
O16—Mo1—O4	84.8 (2)	O21B—O21A—O19B	91.7 (5)
O17—Mo1—O4	85.6 (3)	O19A^i^—O21A—O19B	89.1 (5)
O3—Mo1—O19A	160.1 (3)	P1—O21A—Mo5	123.6 (4)
O13—Mo1—O19A	63.5 (3)	O21B—O21A—Mo5	133.5 (5)
O16—Mo1—O19A	92.7 (3)	O19A^i^—O21A—Mo5	128.7 (5)
O17—Mo1—O19A	64.3 (2)	O19B—O21A—Mo5	68.8 (4)
O4—Mo1—O19A	92.9 (3)	P1—O21A—Mo4	125.0 (4)
O3—Mo1—O19B	157.3 (3)	O21B—O21A—Mo4	70.0 (4)
O13—Mo1—O19B	93.6 (3)	O19A^i^—O21A—Mo4	132.5 (5)
O16—Mo1—O19B	61.9 (3)	O19B—O21A—Mo4	132.6 (4)
O17—Mo1—O19B	92.8 (3)	Mo5—O21A—Mo4	92.4 (2)
O4—Mo1—O19B	63.0 (3)	P1—O21A—Mo3	123.7 (4)
O19A—Mo1—O19B	42.6 (2)	O21B—O21A—Mo3	130.2 (5)
O15—Mo2—O16^i^	103.1 (3)	O19A^i^—O21A—Mo3	68.4 (3)
O15—Mo2—O11	100.5 (3)	O19B—O21A—Mo3	130.5 (5)
O16^i^—Mo2—O11	89.8 (3)	Mo5—O21A—Mo3	91.2 (3)
O15—Mo2—O14	101.5 (3)	Mo4—O21A—Mo3	91.1 (3)
O16^i^—Mo2—O14	155.4 (3)	P1—O19B—O21A	54.6 (3)
O11—Mo2—O14	86.8 (2)	P1—O19B—O21B^i^	54.4 (4)
O15—Mo2—O12	102.8 (3)	O21A—O19B—O21B^i^	91.4 (5)
O16^i^—Mo2—O12	88.3 (2)	P1—O19B—Mo5	124.4 (4)
O11—Mo2—O12	156.4 (3)	O21A—O19B—Mo5	69.7 (4)
O14—Mo2—O12	85.3 (2)	O21B^i^—O19B—Mo5	133.4 (5)
O15—Mo2—O21B	158.5 (3)	P1—O19B—Mo2^i^	124.9 (4)
O16^i^—Mo2—O21B	92.4 (3)	O21A—O19B—Mo2^i^	134.5 (5)
O11—Mo2—O21B	64.3 (3)	O21B^i^—O19B—Mo2^i^	70.5 (4)
O14—Mo2—O21B	64.1 (3)	Mo5—O19B—Mo2^i^	92.5 (3)
O12—Mo2—O21B	92.3 (3)	P1—O19B—Mo1	122.3 (4)
O15—Mo2—O19B^i^	160.6 (3)	O21A—O19B—Mo1	129.3 (4)
O16^i^—Mo2—O19B^i^	63.6 (3)	O21B^i^—O19B—Mo1	130.1 (5)
O11—Mo2—O19B^i^	93.8 (3)	Mo5—O19B—Mo1	91.9 (2)
O14—Mo2—O19B^i^	92.3 (3)	Mo2^i^—O19B—Mo1	91.4 (2)
O12—Mo2—O19B^i^	64.4 (3)	P1—O21B—O21A	56.3 (4)
O21B—Mo2—O19B^i^	40.9 (2)	P1—O21B—O19B^i^	55.9 (4)
O10—Mo3—O1	102.8 (3)	O21A—O21B—O19B^i^	93.1 (5)
O10—Mo3—O7	102.3 (3)	P1—O21B—Mo4	126.2 (4)
O1—Mo3—O7	90.7 (2)	O21A—O21B—Mo4	69.8 (4)
O10—Mo3—O13^i^	101.3 (3)	O19B^i^—O21B—Mo4	134.1 (5)
O1—Mo3—O13^i^	90.7 (3)	P1—O21B—Mo2	124.5 (4)
O7—Mo3—O13^i^	155.4 (3)	O21A—O21B—Mo2	132.1 (5)
O10—Mo3—O9	100.2 (3)	O19B^i^—O21B—Mo2	68.6 (4)
O1—Mo3—O9	157.0 (3)	Mo4—O21B—Mo2	91.4 (3)
O7—Mo3—O9	85.7 (3)	P1—O21B—Mo6	122.3 (4)
O13^i^—Mo3—O9	83.5 (2)	O21A—O21B—Mo6	131.5 (5)
O10—Mo3—O19A^i^	156.4 (3)	O19B^i^—O21B—Mo6	127.9 (5)
O1—Mo3—O19A^i^	95.4 (3)	Mo4—O21B—Mo6	91.6 (3)
O7—Mo3—O19A^i^	92.2 (3)	Mo2—O21B—Mo6	90.9 (3)
O13^i^—Mo3—O19A^i^	63.2 (3)	O21A—P1—O21A^i^	180.0 (7)
O9—Mo3—O19A^i^	62.1 (3)	O21A—P1—O21B	68.6 (4)
O10—Mo3—O21A	161.4 (3)	O21A^i^—P1—O21B	111.4 (4)
O1—Mo3—O21A	65.6 (3)	O21A—P1—O21B^i^	111.4 (4)
O7—Mo3—O21A	64.9 (3)	O21A^i^—P1—O21B^i^	68.6 (4)
O13^i^—Mo3—O21A	93.5 (3)	O21B—P1—O21B^i^	180.0 (6)
O9—Mo3—O21A	92.5 (3)	O21A—P1—O19B	70.5 (4)
O19A^i^—Mo3—O21A	42.1 (2)	O21A^i^—P1—O19B	109.5 (4)
O8—Mo4—O6	103.7 (3)	O21B—P1—O19B	110.3 (4)
O8—Mo4—O11	102.2 (3)	O21B^i^—P1—O19B	69.7 (4)
O6—Mo4—O11	90.1 (2)	O21A—P1—O19B^i^	109.5 (4)
O8—Mo4—O5	100.8 (3)	O21A^i^—P1—O19B^i^	70.5 (4)
O6—Mo4—O5	90.7 (2)	O21B—P1—O19B^i^	69.7 (4)
O11—Mo4—O5	156.1 (3)	O21B^i^—P1—O19B^i^	110.3 (4)
O8—Mo4—O1	99.2 (3)	O19B—P1—O19B^i^	180.0 (6)
O6—Mo4—O1	157.0 (3)	O21A—P1—O19A	108.9 (4)
O11—Mo4—O1	85.4 (2)	O21A^i^—P1—O19A	71.1 (4)
O5—Mo4—O1	84.7 (2)	O21B—P1—O19A	71.1 (4)
O8—Mo4—O21B	162.1 (3)	O21B^i^—P1—O19A	108.9 (4)
O6—Mo4—O21B	65.8 (3)	O19B—P1—O19A	72.2 (4)
O11—Mo4—O21B	64.7 (3)	O19B^i^—P1—O19A	107.8 (4)
O5—Mo4—O21B	94.0 (3)	O21A—P1—O19A^i^	71.1 (4)
O1—Mo4—O21B	92.0 (3)	O21A^i^—P1—O19A^i^	108.9 (4)
O8—Mo4—O21A	157.5 (3)	O21B—P1—O19A^i^	108.9 (4)
O6—Mo4—O21A	93.0 (3)	O21B^i^—P1—O19A^i^	71.1 (4)
O11—Mo4—O21A	92.4 (3)	O19B—P1—O19A^i^	107.8 (4)
O5—Mo4—O21A	63.7 (3)	O19B^i^—P1—O19A^i^	72.2 (4)
O1—Mo4—O21A	64.7 (2)	O19A—P1—O19A^i^	180.0 (7)
O21B—Mo4—O21A	40.2 (3)		

Symmetry codes: (i) −*x*+1/2, −*y*+1/2, −*z*.

### Hydrogen-bond geometry (Å, °)

**Table:**

*D*—H···*A*	*D*—H	H···*A*	*D*···*A*	*D*—H···*A*
N3—H3A···O17^ii^	0.86	1.95	2.803 (8)	171
N6—H6···O2W	0.86	2.25	3.102 (17)	173
N9—H9A···O1W	0.86	1.96	2.790 (11)	163

Symmetry codes: (ii) −*x*+1/2, *y*+1/2, −*z*+1/2.
